# Recording of pig neuronal activity in the comparative context of the awake human brain

**DOI:** 10.1038/s41598-022-19688-2

**Published:** 2022-09-15

**Authors:** Aksharkumar Dobariya, Tarek Y. El Ahmadieh, Levi B. Good, Ana G. Hernandez-Reynoso, Vikram Jakkamsetti, Ronnie Brown, Misha Dunbar, Kan Ding, Jesus Luna, Raja Reddy Kallem, William C. Putnam, John M. Shelton, Bret M. Evers, Amirhossein Azami, Negar Geramifard, Stuart F. Cogan, Bruce Mickey, Juan M. Pascual

**Affiliations:** 1 Rare Brain Disorders Program, Department of Neurology, The University of Texas Southwestern Medical Center, 5323 Harry Hines Blvd. Mail Code 8813, Dallas, TX 75390-8813 USA; 2 Department of Neurological Surgery, The University of Texas Southwestern Medical Center, Dallas, TX 75390 USA; 3 Department of Bioengineering, The University of Texas at Dallas, Richardson, TX 75080 USA; 4 Animal Resource Center, The University of Texas Southwestern Medical Center, Dallas, TX 75390 USA; 5 Department of Neurology, The University of Texas Southwestern Medical Center, Dallas, TX 75390 USA; 6 Department of Pharmacy Practice and Clinical Pharmacology, Experimental Therapeutics Center, Texas Tech University Health Sciences Center, Dallas, TX 75235 USA; 7 Department of Pharmaceutical Science, School of Pharmacy, Texas Tech University Health Sciences Center, Dallas, TX 75235 USA; 8 Department of Internal Medicine, The University of Texas Southwestern Medical Center, Dallas, TX 75390 USA; 9 Department of Pathology, The University of Texas Southwestern Medical Center, Dallas, TX 75390 USA; 10 Department of Physiology, The University of Texas Southwestern Medical Center, Dallas, TX 75390 USA; 11 Department of Pediatrics, The University of Texas Southwestern Medical Center, Dallas, TX 75390 USA; 12 Eugene McDermott Center for Human Growth and Development/Center for Human Genetics, The University of Texas Southwestern Medical Center, Dallas, TX 75390 USA; 13 Present Address: Department of Neurosurgery, Loma Linda University Medical Center, Loma Linda, CA 92354 USA

**Keywords:** Cellular neuroscience, Neural circuits, Neuronal physiology, Synaptic transmission

## Abstract

Gyriform mammals display neurophysiological and neural network activity that other species exhibit only in rudimentary or dissimilar form. However, neural recordings from large mammals such as the pig can be anatomically hindered and pharmacologically suppressed by anesthetics. This curtails comparative inferences. To mitigate these limitations, we set out to modify electrocorticography, intracerebral depth and intracortical recording methods to study the anesthetized pig. In the process, we found that common forms of infused anesthesia such as pentobarbital or midazolam can be neurophysiologic suppressants acting in dose-independent fashion relative to anesthetic dose or brain concentration. Further, we corroborated that standard laboratory conditions may impose electrical interference with specific neural signals. We thus aimed to safeguard neural network integrity and recording fidelity by developing surgical, anesthesia and noise reduction methods and by working inside a newly designed Faraday cage, and evaluated this from the point of view of neurophysiological power spectral density and coherence analyses. We also utilized novel silicon carbide electrodes to minimize mechanical disruption of single-neuron activity. These methods allowed for the preservation of native neurophysiological activity for several hours. Pig electrocorticography recordings were essentially indistinguishable from awake human recordings except for the small segment of electrical activity associated with vision in conscious persons. In addition, single-neuron and paired-pulse stimulation recordings were feasible simultaneously with electrocorticography and depth electrode recordings. The spontaneous and stimulus-elicited neuronal activities thus surveyed can be recorded with a degree of precision similar to that achievable in rodent or any other animal studies and prove as informative as unperturbed human electrocorticography.

## Introduction

Our motivation was to facilitate the characterization of neurophysiological activity in a complex large animal brain as comprehensively as possible (i.e., using several recording modalities that span from individual neurons to the electroencephalogram) and with as little experimental interference or constrains as possible. To this effect, we utilized the pig and assessed the neurophysiological impact of modifying commonly used investigative methods in addition to using novel amorphous silicon carbide microelectrode arrays. Here we report the optimal method as judged from its reproducibility, recording precision and physiological information content. The overall objective was to achieve comparability of neurophysiological activity using the human brain as a standard. Besides aiming for wide applicability, this work is informed by four scientific considerations, each leading to a scientific goal of the study.

The first consideration is comparative. Studies of gyriform brains have been prominent throughout the history of neuroscience^1^. With the advent of transgenic methodology, the laboratory mouse came to dominate much neurophysiological research applied to both physiology and pathophysiology. However, there have been public calls by patient advocacy and professional societies in search of more robust neurological research models since it is broadly recognized that biomedical brain research in rodents and simpler organisms is inevitably constrained by limited applicability to treatment development. The pig is thus desirable as a model because it circumvents several biological disadvantages relative to other models^2^.

A second consideration is the effect of anesthetics, which can suppress neurophysiological activity via an increasingly understood array of mechanisms. Except perhaps for interference with neurotransmitter cycling^3^, the anesthetic effects of most clinically-relevant agents on lipids^4^ and cortical neurons^5^ are nonlinear relative to anesthetic concentration and thus cannot be quantitatively anticipated or otherwise accounted for other than experimentally^6^. This experimental observation requirement is also supported by the fact that pharmacological modulation of a neural network by neuroactive drugs may be, paradoxically, network area dependent^7^. Thus, we reasoned that comparative analysis of neurophysiological activity with the awake human brain would provide an appropriate standard.

Third, much regional neurophysiological activity in the intact brain such as the electroencephalogram (EEG) or electrocorticogram (ECoG) is oscillatory, but the oscillation periodicity nears that of the alternating electric current that powers recording and other laboratory instruments. For example, inhibitory neurons give rise to high frequency oscillations, of which those greater than 40 Hz in periodicity are of particular importance in some disease states^8^. Yet, electric current noise oscillates predominantly at 50–60 Hz and can thus interfere with recording. Low noise EEG and ECoG recordings are valuable, as it is not possible to infer EEG or ECoG oscillations from neuronal oscillations, particularly since the recording of spontaneous EEG or ECoG activity is not necessarily correlated with intracerebral neuronal activity, as the former is more vulnerable to certain types of clinically relevant injury than the latter. For example, in some experimental preparations, there is often preservation of cellular activity measured as local field potentials while there is also cessation of the ECoG signal^9^. Therefore, noise reduction is desirable to capture all potential signals.

Lastly, microelectrodes used to record single-neuron activity may cause significant tissue disruption. Often, optimal recordings require a stabilization period of several days post implantation, which is assumed to stem from tissue recovering its structure^10–12^. Thus, thin, high-density microelectrode probes made of amorphous silicon carbide with the capacity to simultaneously record from multiple areas would represent an advantage^13,14^.

We illustrate these four aspects as applied primarily to the cerebral cortex. The cortex is particularly informative in disease models because (a) it is the best characterized and accessible area for neurophysiological analysis, including in metabolic brain disease^8,15^, (b) its cell types and synaptic and metabolic activities are well delineated, (c) its transcriptome has been characterized, (d) it represents a clinically relevant area both from a symptomatic (including post stroke seizures^16^) and human EEG monitoring point of view, thus enabling the evaluation of therapy^17^ and (e) cortical lesions of predictable dimension are reliably achieved in the pig^18^.

We also aimed to simultaneously acquire intracortical and global (sometimes called macroscopic) cerebral activity to enable robust descriptions of normal and disease states at complementary biological levels of observation because metabolic or pharmacological treatments can modulate specific cell type function and because the evaluation and treatment of networks benefits, as exemplified by epilepsy, from characterizing local circuit properties not apparent from network data. Thus, our method aimed to enable generating an overview of neurophysiological mechanisms across the brain and a characterization of synaptic function in the cortex of a mammal functionally relatively close to man.

## Methods

### Animals

The animal portion of the study was approved by the Institutional Animal Care and Use Committee of UT Southwestern Medical Center and all other relevant institutional guidelines and regulations were followed, in addition to ARRIVE (Animal Research: Reporting of In Vivo Experiments) guidelines. 17 domestic farm pigs (*Sus scrofa domesticus*, Yorkshire cross) including approximately equal number of males and females were studied. There was no significant difference in any of the physiological monitoring or neurophysiological parameters measured between males and females and thus the results are reported regardless of sex. We studied juvenile pigs of 4–5 months of age weighing 20–25 kg because of progressive limitation to surgical intracranial accessibility with age. For reference, by 5.5 months, the pig behavioral repertoire is full and brain weight reaches about 95% of maximum^19^. We noted that, in older pigs obtained from the same source, the skull continued to grow in the craniocaudal direction by more than 2.5 cm at the cranial apex (n = 7 pigs aged > 6 months), rendering the surgical approach less practicable.

Pigs were acquired 1–4 weeks before study and maintained at an Association for Assessment and Accreditation of Laboratory Animal Care International accredited UT Southwestern facility. They were pair- or group-housed in elevated pens on tenderfoot or slatted flooring compliant with the space characteristics outlined in the Guide for the Care and Use of Laboratory Animals. Housing was temperature and humidity-controlled at 18–22 °C and 30–70%, respectively and maintained on a 12:12 h light–dark cycle. They were fed a commercial diet (Teklad Mini swine Diet 7037, Envigo, Madison, WI) and provided with ad-libitum water via an automated system.

### Anesthesia

About 30 min prior to surgery, pigs were sedated with an intramuscular injection of tiletamine and zolazepam (4–8 mg/kg of each, in equal amount), atropine (0.04 mg/kg) and buprenorphine (0.05 mg/kg). They were then administered inhaled isoflurane (1–5%) and oxygen (2 L/min) via face mask, followed by endotracheal intubation, intravenous and intraarterial catheter and esophageal EKG probe and rectal temperature probe insertion. An intravenous saline infusion was maintained at 50 ml/h. The respiratory rate was mechanically assisted as needed with a respirator to maintain 15–20 breaths per minute. General anesthesia was maintained using isoflurane at 1–3% v/v with air (except where noted) and oxygen (2 L/min) applied via the endotracheal tube and mechanical ventilation. Alternatively, where noted, some pigs received intravenous anesthesia with midazolam. In these cases, an initial dose of 0.5 mg/kg was followed by infusion at 0.5–2 mg/kg/h. This was complemented in some cases with additional midazolam doses (1 mg/kg) upon increases in heart rate greater than 10 beats per minute. Where stated, pigs received pentobarbital at 20 mg/Kg/hr. In order to the determine the suitability of several anesthetics for the acquisition of neurophysiological recordings with minimal disruption, we performed recordings under intravenous midazolam anesthesia in 3 pigs and under pentobarbital anesthesia in another. The purpose of this evaluation was not to determine the pharmacodynamic profile of these anesthetics in relation to neurophysiological suppression, which would require further work, but to identify if a potential dose-independence of anesthetic action existed and whether these nonlinearities were sufficiently predictable to fully account for their neurophysiological impact. To this end, ECoG was recorded at increasing, activity suppressing concentrations of midazolam or pentobarbital. Midazolam concentrations of 0.5, 1, 2, and 3 mg/kg/h were compared in terms of the mean power spectra of control recordings under isoflurane anesthesia.

### Animal preparation, monitoring and termination

Heart rate, arterial blood pressure, respiratory frequency, pulse oximetry and capnography were monitored to ensure stability within normal clinical veterinary ranges. The position of the monitoring and recording equipment is illustrated in Supplementary Fig. 1. The anesthetized pig was placed in a prone position on the operating table. A strap was wrapped around the torso and attached to the table to stabilize the body and avoid movement during exposure and craniectomy. The body of the animal was covered with a heated air blanket (Bair hugger, 3M, MN) and the head was surgically draped to allow cranial exposure. Procedures performed in each subject is laid out with a flowchart in Supplementary Fig. 2. To reduce electrical noise, we designed a Faraday cage using copper mesh supported by a wood frame structure (Supplementary Fig. 3). All recording and electrical stimulation procedures took place inside the cage, which was maintained closed during recording unless otherwise stated.

Euthanasia was induced at the conclusion of the study while under general anesthesia by the intravenous addition of pentobarbital at excess dose (120 mg) to produce asystole, cessation of spontaneous respiration and the development of fixed and dilated pupils with absent corneal reflexes. Necropsy was performed to verify macroscopic electrode placement and brain configuration and integrity.

### Pig electroencephalography

In some pigs, 8 needle electrodes (0.5″ long 27G subdermal needle electrodes, Cadwell Industries, Inc., WA) were subcutaneously inserted before craniectomy in pairs, situated 3 cm symmetrically across the midline and connected to the clinical amplifier described below. A ground electrode was subcutaneously inserted in the dorsal neck. The EEG was recorded for 2 h, followed by electrode withdrawal.

### Craniectomy

**Figure 1:**
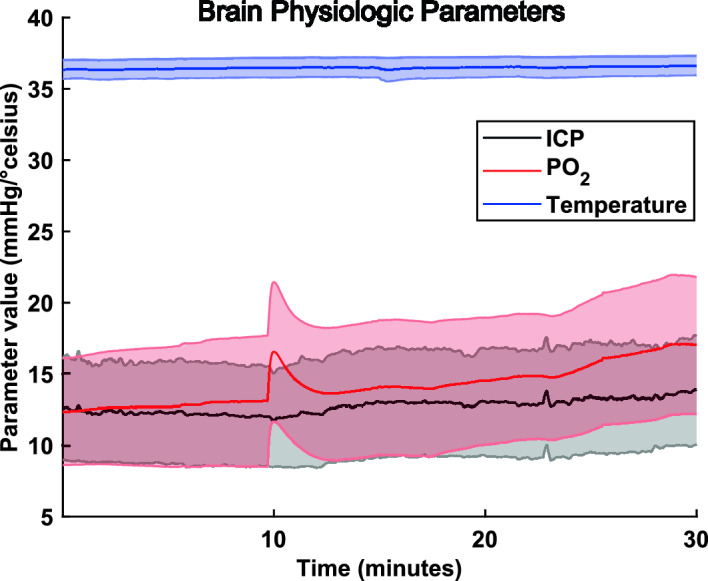
Cerebral viability under anesthesia. Barometric pressure (blue spectra), partial pressure of oxygen (red spectra) and temperature (blue spectra) in the brain of 10 pigs for a period of 30 min. The traces represent mean (dense color lines) and standard deviation ranges (same but lighter color lines).

**Figure 2:**
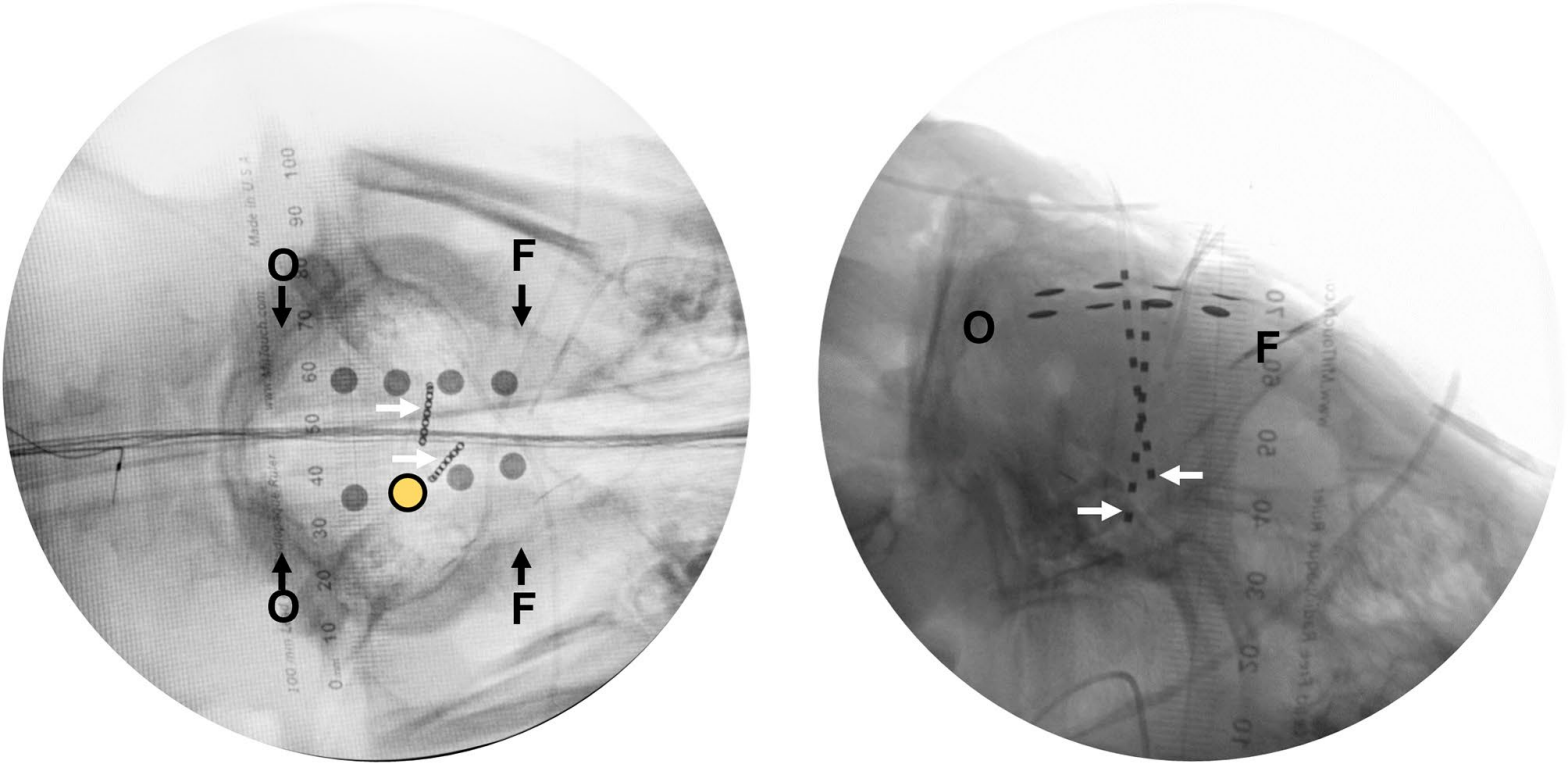
Pig electrode position. Selective views. Craniocaudal (i.e., vertical, left) and lateral (right) X-rays of 8 electrocorticography (in two strips) and 8 depth recording electrodes (in two linear depth electrodes) in place. Two linear, near vertical depth electrodes (8 recoding sites each) are marked with white arrows on each side of the midline. Eight circular pial electrocorticography electrodes are also shown. The highlighted electrode (yellow) was chosen for analysis of comparing activity with human data. Occipital (O) and frontal (F) areas are also marked for orientation. The ruler shown is divided in mm.

After the induction of general endotracheal anesthesia (identified as a state of absence of response to noxious stimuli with minimal variability in vital signs), the pig was placed in the prone position on the operating table. The custom-made Faraday cage placed on the table remained open anteriorly at this point to provide unobstructed access to the head. The surgical field and the instruments were prepared using a clean but not sterile technique. A longitudinal midline incision was marked from a point 3 cm caudal to the external occipital protuberance to the nasion, 14–16 cm in length. The incision was opened with the cutting cautery down to bone. The pericranium was reflected laterally with the scalp as far as the superior temporal line on either side and a self-retaining retractor was placed. Mild hyperventilation was initiated to minimize the likelihood of cortical bruising during the performance of the craniectomy. A burr hole was placed with a power perforator 1.5 cm on either side of the midline at the level of the coronal suture. Using a craniotome and rongeurs, an oval craniectomy, 5 cm rostral-caudal and 4 cm transverse was created. The caudal extent of the craniectomy was 2 cm anterior to the superior nuchal line. (Supplementary Fig. 4). The dura was opened in a flap based medially on either side of the midline, taking care to avoid injury to the bridging veins and the superior sagittal sinus. Strip or grid electrodes (Ad-tech, Il) were placed over the cerebral convexity bilaterally, parallel to and 1 cm lateral to the superior sagittal sinus. One depth electrode (Ad-tech) was placed on each side of the midline at the level of the coronal suture to capture deep brain activity. A Neurovent-PTO 2L Brain probe (Raumedic, Germany) was inserted into the left frontal lobe at a depth of 1.5 cm for temperature, intracranial pressure, and tissue oxygen saturation monitoring (Fig. 1). After securing the electrodes and brain probe, the scalp was closed with sutures. The positions of the electrodes and brain probe were documented radiographically using craniocaudal and lateral views (Fig. 2).

### Brain tissue anesthetic concentrations

A 14-gauge brain needle biopsy with 8 mm window opening was used to extract cerebral cortical samples weighing approximately 15 mg. The samples were immediately freeze clamped on dry ice and stored in liquid nitrogen. These brain biopsies were used for determining the brain concentration of midazolam and pentobarbital as described below.

### Midazolam bioanalytical method

Midazolam and Midazolam-d4 certified reference material (CRM) in methanol was purchased from Cayman chemicals (Ann Arbor, MI). Midazolam plasma and brain concentrations were determined using a UHPLC-MS/MS analytical method that included a Shimadzu LC-30AD Nexera Series system (Shimadzu Corporation, Japan) and a Sciex 6500 + QTRAP mass spectrometer (Foster City, CA) that was equipped with a Turboionspray™ interface. Pre-weighed brain samples were homogenized using 4 volumes of LC–MS grade water on a DPS-20 dual processing system from Pro Scientific Inc. (Oxford, CT). Sample preparation included extraction with methyl tert-butyl ether and methylene chloride (3:1, v/v), in the presence of midazolam-d4 internal standard (IS). The method was validated in both pig plasma and pig brain homogenate using a Phenomenex Kinetex XB C18 100 Å, 2.6 µm, 50 × 2.1 mm (Phenomenex, CA) column and UHPLC C18 2.1 mm guard column. Gradient elution was employed with mobile phase A (MP-A) consisting of 0.1% formic acid in water and mobile phase B (MP-B) consisting of 0.1% formic acid in methanol. The chromatographic flow rate was 0.4 mL/min and a 10 µL injection volume was used. Detection of midazolam and IS was carried out in the multiple-reaction monitoring mode (MRM), by monitoring the transition pairs of m/z 326.3 (precursor) to m/z 291 (product) for midazolam, 342.3–203 for 1-hydroxy midazolam, and 330.0–295.1 for midazolam-d4. The data were processed with Analyst software™ (version 1.7.1).

### Pentobarbital bioanalytical method

Pentobarbital and pentobarbital-d5 certified reference material (CRM) in methanol were purchased from Cayman chemicals (Ann Arbor, MI). Pentobarbital (PB) plasma and brain concentrations were determined using a UHPLC-MS/MS analytical method with the same instrumentation as the midazolam method. Pre-weighed brain samples were homogenized using 4 volumes of LC–MS grade water on a DPS-20 dual processing system from Pro scientific Inc. Sample preparation included protein precipitation with acetonitrile in the presence of pentobarbital-d5 internal standard (IS). Chromatographic separation was achieved using a Phenomenex Kinetex XB-C18 analytical column (2.6 µm, 100 Å, 2.1 × 50 mm) at 40 ± 2 °C. Gradient elution was employed where mobile phase A (MP-A) was 0.1% formic acid in water and mobile phase B (MP-B) was 0.1% formic acid in methanol. A flow rate of 0.4 mL/min and a 5 µL injection volume were used. Detection of PB and IS was carried out in the positive ion mode utilizing electrospray ionization (ESI) MRM, by monitoring the precursor to product ion transition pairs of 225.1–182.1 m/z for PB, and 230.1–187.1 for IS. The analytical data obtained were processed using Sciex Analyst software™ (version 1.7.1).

### Neurophysiological recording

#### Pig electrocorticography

The strip or grid electrodes were connected, together with a needle ground electrode inserted subcutaneously in the dorsum of the neck, to a clinical 32-channel amplifier (Neurofax EEG-1200, Nihon Kohden, Japan). The ECoG recording parameters are listed in Supplementary Table 2. Recording was continuous for 2–10 h. Several minute intervals were selected for analysis (when needed after band-stop filtering of 59–61 Hz to reduce powerline noise) as noted below after visual inspection of the entire recording. Recording sampling rate was 1000 Hz, band-pass filtered at 0.5–300 Hz.

#### Pig depth electrode recording

Two linear electrodes each containing 8 platinum cylindrical recording sites were inserted in the craniocaudal (vertical) direction. Recording sampling rate was 1000 Hz and a band-pass filter of 0.5–300 Hz was applied.

#### Human electrocorticography

**Figure 3:**
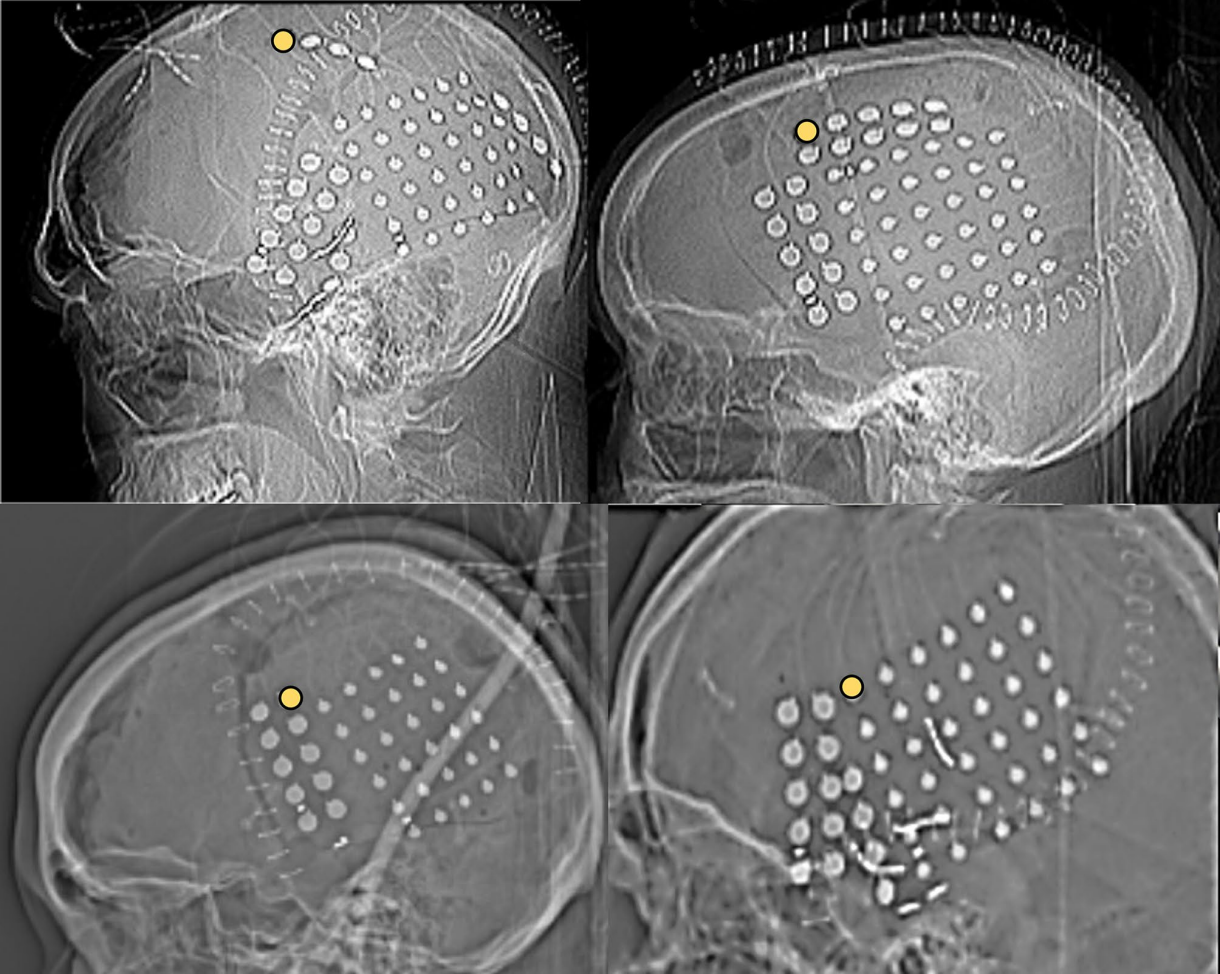
Human electrode position Lateral radiographic images of human electrocorticography grid and strip position. The circular recording electrodes are discernible from the curvilinear surgical staples. Highlighted electrodes (yellow) were selected for power compassions with the pig.

The human part of the study was approved by the Institutional Review Board (IRB) of UT Southwestern Medical Center and all other relevant institutional guidelines and regulations were followed. This included the exemption of informed consent for this study by the IRB since a procedure was put in place to de-identify subject information for the investigators and no personal identifiable data were used. Recordings from human subjects who underwent long-term video grid ECoG monitoring for clinical diagnostic purposes in an inpatient Epilepsy Monitoring Unit were selected. They were implanted with the same type of grid and strip electrodes (59–104 electrodes total) placed in different configurations to cover a large extent of the frontal, parietal, or temporal brain surface (Fig. 3). Concurrent continuous pulse oximeter (SpO2) readings were collected from a Rad-7 oximeter (Masimo, CA), which was connected to the EEG data acquisition system (Nihon Kohden), together with the output of single-channel electrocardiography with the two leads placed across the shoulders. The EKG and SpO2 data were verified at least hourly and were normal during the recordings analyzed. The subjects were selected from our institutional clinical database (containing more than 100 subjects) based on their normal ECoG and absence of lesions detectable by magnetic resonance imaging in the area directly below the ECoG grids. All patients were awake during the recordings selected for analysis. Some of the patients were receiving one to three anticonvulsant medications during the recording. However, comparative analysis of ECoG across all subjects revealed no significant differences in these subjects relative to unmedicated patients and thus all of them are presented in aggregate fashion.

#### Intracortical recording using standard and silicon carbide electrode arrays

Two types of intracortical microelectrode arrays were inserted into the pig cortex for single-unit recordings: (1) a standard 50 µm thick single shank Michigan-style array with 16 recording sites with 100 µm interspacing (A1×16-3mm-100-177-CM16LP, NeuroNexus Technologies, MI) and (2) an 8 µm thick (240 µm^2^ cross-sectional area at the broadest portion of the shank and 80 µm^2^ at the narrowest), four-shank amorphous silicon carbide (a-SiC) ultrasmall microelectrode array with 32 recording sites (University of Texas at Dallas). Both types of microelectrode arrays were placed around the ECoG strips, targeting the somatosensory cortex (mid Brodmann area 1 human equivalent) up to a depth of 2.2 mm, encompassing cortical layers LII-VI. Devices were mounted onto a micro inserter (NeuralGlider, Actuated Medical Inc., PA) attached to a commercial stereotaxic arm situated on a custom-built frame and inserted at a speed of 0.1 mm/s, and remained tethered to the arm for the entire duration of the recording (procedure adapted from^14,20^). The pia mater was left intact to minimize hemorrhaging due to its dense vasculature^21^, which could be disrupted during electrode insertion^22^. Thus, ultrasonic micro-vibration was used during insertion for pia mater penetration of a-SiC microelectrode arrays, which was needed due to the thin and flexible characteristics of ultrasmall a-SiC electrode arrays. Once inserted, reference and ground wires were placed between the resected dura mater and brain tissue without penetrating.

Intracortical recordings were obtained using a Cereplex Direct acquisition system (Blackrock Microsystems, Inc., UT) with a sampling rate of 30 kHz. Spiking activity was analyzed offline using Plexon Offline Sorter (Plexon Inc., TX). First, a high-pass Butterworth filter of order 4 with a cut-off frequency of Fc = 250 Hz was applied. Then, a threshold of − 4 times the standard deviation of the root-mean-square baseline noise was used to detect individual action potentials (spikes; waveform duration = 1.8 ms, pre-threshold period = 0.4 ms, refractory period = 1.33 ms). Single-unit sorting was performed using a K-means scan (K = 2–4) and then manually validated to exclude non-neuronal activity. Waveforms, raster plots and spiking activity were analyzed in Neuroexplorer (Nex Technologies, CO).

A paired-pulse stimulation protocol was used to assess evoked synaptic activity. For recording, we used a single tungsten microelectrode of 75 µm diameter and a final taper angle of 25° with an impedance of 2MΩ (UEWLCESE2NNM, FHC Neural Microtargeting Worldwide, ME). For stimulation, we used a Tungsten Concentric Bipolar microelectrode with a 2–3 µm tip diameter (TM33CCINS, FHC Neural Microtargeting Worldwide). Both electrodes were attached to the handle of a cotton tip applicator and inserted using a stereotaxic arm. The distance between the recording and stimulating electrodes was less than 1 mm. We recorded a baseline for 30 s, followed by six biphasic pulses (pulse width = 200 µs, interphase delay = 5 µs) at two different pulse rates: 10 and 20 Hz; and two amplitudes: 20 and 40 µA. Evoked activity was analyzed offline following the procedure described above.

### Neurophysiological data analysis

#### Power spectral density analysis

All ECoG and depth electrode figures represent relative power spectral density. Parietal grid electrodes were selected from pigs (R3) and human subjects (example electrode contacts are in Figs. 2 and 3) for the calculation of power spectral density (PSD). This included oscillation frequencies in the range of delta, theta, alpha, beta and gamma range of frequencies. Welch’s method of fast Fourier transform was utilized to calculate power for each frequency level^23^. Supplementary information includes detailed code to calculate power. The mean and standard error of the power spectra was calculated using MATLAB’s *smooth* function. We used ‘*shadedErrorBar’* github code to plot mean and standard error values of the spectra^24^. Supplementary Fig. 5 depicts the power spectral density algorithm in flow chart format.

#### Neurophysiological coherence analysis

Parietal grid electrodes symmetrically situated in both hemispheres were selected for the calculation of coherence. The supplementary information contains the code used for coherence analysis. These plots are presented smoothed as mean and standard error as derived from ‘*shadedErrorBar*’. Supplementary Fig. 5 depicts the coherence algorithm as a flow chart.

#### Transfer function calculation

Scalp EEG and ECoG data were used to calculate a transfer function to better illustrate loss of power in the former recordings. We utilized MATLAB’s ‘*tfestimate*’ to calculate the function results presented in Supplementary Fig. 6.

### Histological analysis

Five pigs were utilized to measure cerebral cortex thickness and layer organization using standard formalin fixation and paraffin histologic processing. Resulting coronal sections were stained with Nissl/luxol fast blue, as well as Nissl/Periodic Acid Schiff histochemical stains according to established methods. 5 coronal sections of cortex and subjacent white matter including frontal, parietal, occipital and temporal cortices were studied in each pig. Cortical layers were resolved aided by the staining and identified by comparison with the layering of the human cortex for the equivalent regions. The sections were photographed at low and intermediate objective magnifications on a Leica DM2000 research microscope equipped with a Jenoptik Gryphax NAOS CMOS camera for comparison to intracortical recordings. The comparison utilized the depth of microelectrode penetration, which was recorded by video, with post hoc histological sections of the recorded area.

### Ethical approval

*Guidelines followed* ARRIVE (Animal Research: Reporting of In Vivo Experiments) and all applicable guidelines and regulations of UT Southwestern Medical Center. *Animal use approval* Institutional Animal Care and Use Committee of UT Southwestern Medical Center. *Human subjects research approval* Institutional Review Board of UT Southwestern Medical Center.

## Results

### Pig cerebral viability

Animals were stable (i.e., within 30% of initial values for all physiological parameters) for at least 9 h after anesthesia induction. Figure 1 illustrates temperature, intracerebral pressure and oxygen pressure in 10 pigs during the entire anesthesia period, including the intervals used for neurophysiological recording. Mean temperature was 36.6 °C, mean intracerebral pressure was 12.9 mmHg and mean oxygen pressure was 13.9 mmHg, with no significant deviation over the course of the recording. These viability values are similar to reported data^25^, considering that pig intracerebral pressure and intracranial pressure are affected by removal of the cranial vault.

### Pig electrocorticography and depth recording

**Figure 4:**
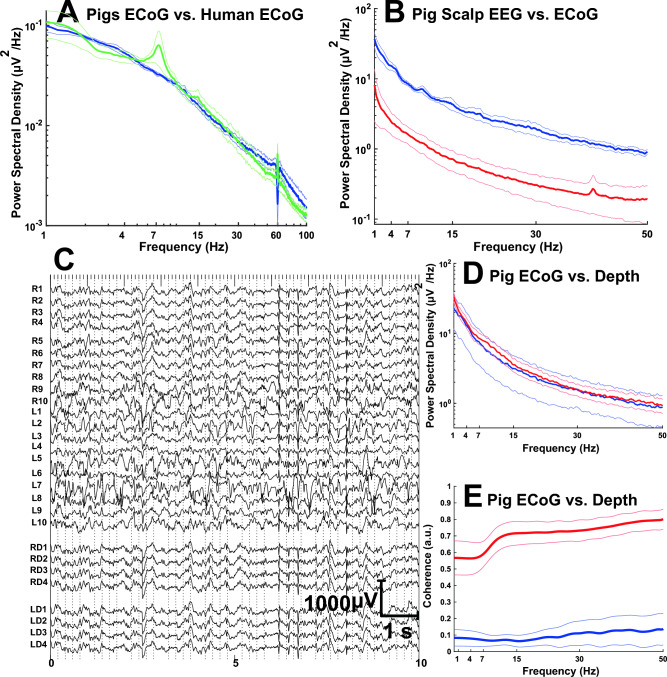
Human and pig neurophysiological network recordings. Pig electroencephalography, electrocorticography and depth electrode recordings, and human electrocorticography compared. (**A**) Human and pig ECoG comparison. Mean and standard error coherence of pig ECoG (blue line) and human ECoG (green line) recordings. (**B**) Mean (central line, intense color) and standard error (surrounding lines, less intense same color) power spectral density spectra of pig ECoG (blue line) and pig EEG (red line) recordings. (**C**) Unmodified pig ECoG (R1–R10, right and L1–L10, left ECoG electrodes) and depth electrode (RD1–RD4, right and LD1–LD4, left) tracings. (**D**) Pig mean and standard error power spectral density spectra of ECoG (blue) and depth (red) recordings, n = 6 pigs. (**E**) Pig mean and standard error coherence of ECoG (blue) and depth (red) recordings measured at electrodes R1-L1 and RD1-LD1 with n = 6 pigs. 1 indicates complete coherence, whereas 0 reflects absence of coherence.

Figure 4 illustrates recordings made in one pig anesthetized with 1.5% isoflurane from 10 surface and 4 depth electrodes in each hemisphere, covering frontal, temporal, parietal, and occipital lobes in addition to subcortical brain regions including white matter, and the vicinity of the striatum and hippocampus (a partial preparation is shown in Fig. 2). Figure 4 also displays representative power spectral density and coherence spectra from electrocorticography and depth recording from this and an additional five pigs. The power spectrum of the ECoG and depth recordings was unchanged throughout the duration of each study, which span from about 7–10 h depending on each pig. However, coherence varied between ECoG and depth recordings, with depth recordings exhibiting greater coherence than ECoG as it is also noticeable from the recording traces in Fig. 4.

Neurophysiological recordings obtained by us in two standard laboratories were hampered by electrical power artifact. Analysis of this noise revealed an unpredictable periodicity oscillation of elevated amplitude around 60 Hz, masking important frequencies in the gamma range, which are indicators of inhibitory neuron activity as supported by poison inhibition of the respiratory chain, which abolishes EEG gamma oscillations^26^. These gamma oscillations can be used to predict human seizures in human and mouse metabolic brain disease^8^. Using a copper Faraday cage (Supplementary Fig. 3) to which the pig was grounded, this was mitigated except for the persistence of a narrower noise band at ~ 60 Hz (Supplementary Fig. 7). Under the cage, only a small window (59–61 Hz) of the recordings continued to exhibit significant noise. Thus, the interpretation of data remained impractical for that residual frequency range.

### Human and pig neurophysiological comparison

Figure 4 illustrates the averaged ECoG of 4 awake human subjects and 5 anesthetized pigs recorded from the parietal lobe represented as power spectral density and coherence. The power spectra of the pig resembled the normal human ECoG power spectrum, with relative prominence of low to mid-range of frequencies (1–15 Hz) and diminished power at high and very high ECoG frequencies (35–100 Hz). A 7–10 Hz frequency characteristic of conscious subjects engaged in visualization was present in the human recordings. As apparent from Fig. 4A, human low alpha activity (7–9 Hz) was significantly different when compared with the pig alpha activity (Mann–Whitney non-parametric t-test, *p* = 0.0159).

### Pig electroencephalography and electrocorticography comparison

We compared signals recorded from pig scalp EEG with ECoG under 1.5% isoflurane anesthesia in 5 pigs. As illustrated in Fig. 4, the entire EEG frequency spectrum was characterized by decreased power compared to ECoG. Unpaired Welch’s t tests showed significantly lower power for Delta (*p* = 0.0128), Theta (*p* = 0.0006), Alpha (*p* = 0.0032), Beta (*p* = 0.0036) and Gamma (*p* = 0.0011) activities for scalp EEG relative to ECoG. This is likely due to distance from source, impedance and other factors that result in degraded EEG signals relative to ECoG. Further analysis of these two types of activity using transfer function revealed active frequency independent dampening of EEG signals by scalp as the amplitude was much lower for EEG (Supplementary Fig. 6). For 0.1–100 Hz, the dampening effect was slightly greater than other frequencies and decreased until 200 Hz, followed by consistent dampening beyond 200 Hz (Supplementary Fig. 6b).

### Suppressant effects of anesthetics in the pig brain

**Figure 5:**
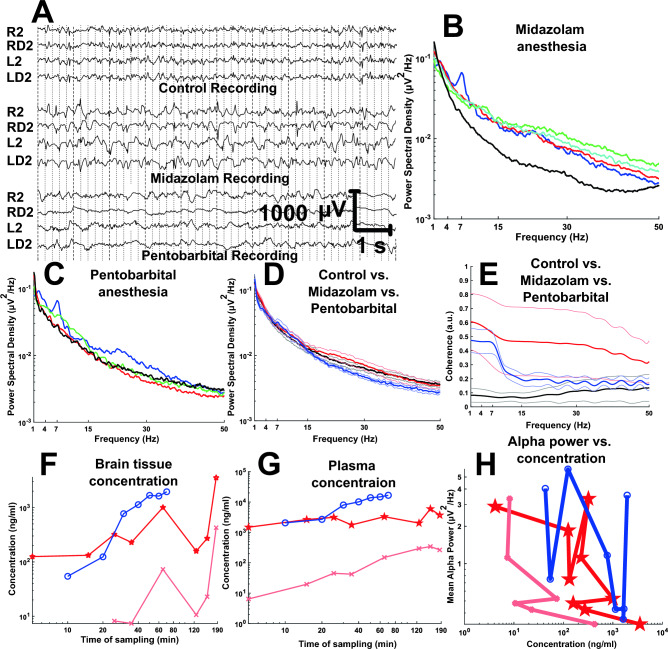
Neurophysiological effects of intravenous anesthetics. Midazolam, Pentobarbital and Isoflurane (control) anesthesia ECoG. (**A**) ECoG traces of control, midazolam and pentobarbital anesthesia. (**B**) Power spectra of control (blue line), 0.5 mg/Kg/h (red line), 1 mg/Kg/h (green line), 2 mg/Kg/h (cyan line), 3 mg/Kg/h (black line) Midazolam IV infusion. (**C**) Power spectral of control (blue line), 20 mg/kg/h (red line), 40 mg/kg/h (green line), 60 mg/kg/h (black line) Pentobarbital IV infusion. (**D**) Mean and standard error of control (black line), midazolam (red line), and pentobarbital (blue line) power spectra. (**E**) Mean and standard error of control (black line), midazolam (red line), and pentobarbital (blue line) coherence spectra. (**F**) Brain tissue mean concentration relative to time of sampling of pentobarbital (o labels and blue line), hydroxy- (OH)-midazolam (x labels and light red line), midazolam (pentagrams and red line). in infused subjects. (**G**) Plasma mean concentration versus time of plasma sampling of pentobarbital (o labels and blue line), OH-midazolam (x labels and light red line), midazolam (pentagrams and red line). (**H**) Mean alpha power comparison with the increase in brain mean tissue concentration of pentobarbital (o labels and blue line), OH-midazolam (x labels and light red line), midazolam (pentagrams and red line).

We did not observe a gradual concentration dependence to the progression of brain activity suppression. The highest midazolam dose (3 mg/Kg/hr) suppressed power disproportionately relative to the lower concentrations. Midazolam also exerted a widely variable impact on coherence for the same concentration and time of use in different pigs. Similarly, pentobarbital was continuously infused at 20, 40, and 60 mg/Kg/hr without a concentration dependent impact (Fig. 5). Control recordings under 1% isoflurane, which is generally not considered a significant EEG suppressant at low concentrations^27^, exhibited a distinct alpha peak which was suppressed using midazolam or pentobarbital anesthesia. These midazolam and pentobarbital effects were not linearly accounted by their brain tissue concentration, including that of the midazolam metabolite hydroxymidazolam. Analysis of blood and brain samples collected over a period during continuous infusion of these agents indicated that pentobarbital concentration in plasma and brain increased nonlinearly, as was also the case for midazolam, particularly with respect to brain tissue concentration (Fig. 5). The neurophysiological repercussions of these drug concentrations were poorly predictable. For example, when the mean alpha power was analyzed relative to brain pentobarbital and midazolam concentration, there was also a non-dose dependent correlation, except at elevated dose, where midazolam suppressed the alpha activity effectively for the duration of the recording. After these observations we deemed pentobarbital and midazolam unsuitable for our purposes until further work is conducted.

### Dose dependent effect of isoflurane anesthesia in the pig

**Figure 6:**
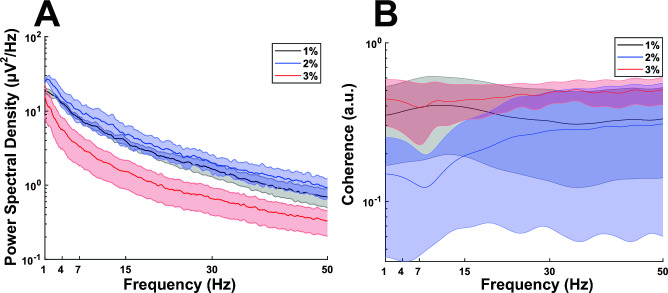
Neurophysiological effects of isoflurane. Dose dependent anesthetic effect of isoflurane general anesthesia on the ECoG. (**A**) Mean and standard error of the power spectral density recorded under 1% isoflurane (black spectra with error lines), 2% isoflurane (blue spectra with error lines) and 3% isoflurane (red spectra with error lines). (**B**) Coherence under 1% isoflurane (black spectra with error lines), 2% isoflurane (blue spectra with error lines) and 3% isoflurane (red spectra with error lines).

Isoflurane is widely used in human surgery due to its brief half-life^28^, which facilitates recovery upon inhalation discontinuation. Isoflurane, however, also suppresses neurophysiological activity at elevated concentrations^29^. We measured the dose dependence of the anesthetic depression of ECoG power and coherence relative to isoflurane concentration in 3 pigs. ECoG epochs were extracted from spatially equivalent left and right-side recordings of the pig brain under 1% (n = 3), 2% (n = 2) and 3% (n = 3 pigs) inhaled isoflurane. In them, we analyzed effects on power and coherence. Similar ECoG power was observed at 1% and 2% isoflurane concentrations (Fig. 6). 3% isoflurane reduced ECoG power as also shown in Fig. 6A. However, the changes were not statistically significant (Mann–Whitney t-test) due to variability across pigs, but also to variability for each pig that was dependent on the sequence of isoflurane concentration changes. This is interesting but merits further investigation since this was not the primary objective of our study.

### Intracortical recording and paired-pulse stimulation

**Figure 7:**
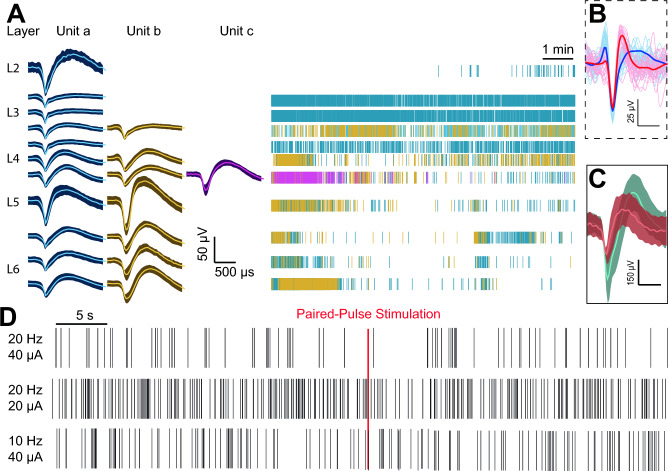
Intracortical recordings. Single-units (i.e., single neuron activities) were recorded across cortical depth. (**A**) Left: Waveforms of up to 3 single-units isolated in a depth-recording using a standard microelectrode array (scale bars = 50 µV and 500 µs). Right: Spike-raster with colors matching single-units on the left (scale bar = 1 min). (**B**) Dotted inset: An example of a narrow-spike (pink) and broad-spike (blue) single-unit (scale bars = 25 µV and 500 µs). Dark lines reflect the average waveform. (**C**) Examples of waveforms isolated from intracortical depth-recordings using amorphous silicon carbide (a-SiC) ultrasmall microelectrode arrays (scale bars = 150 µV and 500 µs). (**D**) Spiking activity during baseline and after paired-pulse stimulation using different pulse frequencies and current amplitudes (scale bar = 5 s).

In order to estimate the depth of layers to record from in intracortical recordings, we measured cortical thickness (Supplementary Figs. 5 and 6). We compared intracortical recordings between a standard microelectrode array and the a-SiC ultrasmall microelectrode array. First, as shown in Fig. 7A, we detected single-unit activity corresponding to the different cortical layers between LII-LVI of the pig somatosensory cortex. Multiple electrode sites corresponding approximately to layers LIII/IV to LVI were able to record 2–3 individual neurons, with some showing waveform characteristics, specifically the spike trough-to-peak duration, that indicate different cell types^8^. Whereas most single units detected appeared to be broad-spikes (putatively pyramidal cells), others (Fig. 7B, red) appeared to be narrow-spikes (inhibitory neurons). The average peak-to-peak amplitude recorded was 37 µV (range 32–57 µV). In contrast, the a-SiC ultrasmall microelectrode array had an average peak-to-peak amplitude of 352 µV (range 133–511 µV). Finally, the paired-pulse stimulation protocol appeared to change the firing rate at 20 Hz but not at 10 Hz with current amplitudes of at least 20 µA. It appears that such electrical stimulation results in a transient (approximately 5 s) firing rate decrease; however, the use of 40 µA resulted in a complete pause of activity. These results demonstrate the feasibility of conducting paired pulse stimulation studies in the pig.

## Discussion

We have developed an approach to study the pig brain that overcomes comparative limitations inherent to small animals. These disadvantages include a paucity of cerebral white matter (which occupies a small fraction of the rodent brain but comprises over 60% of the human or pig brain), a smooth or agyric brain surface configuration, an excessively small number of brain surface neurons relative to depth neurons, a different blood flow recanalization pattern and response to injury^30^, impractical anesthesia or vital function parameter monitoring^31^ and distinct response of the organism to brain disease and injury that deviates from what is characteristic for man and larger mammals. The results presented help establish the suitability of the pig brain as a neurophysiological testbed and its similarity with the activity of the human brain. The principal advantages of the methodology described as applied to the pig are the capacity to study, immediately upon electrode positioning, short and long-term (several hours) events and to experimentally manipulate the brain.

Technical obstacles such as electrical noise in the range of high frequency neural oscillations can be reduced. This is valuable because gamma oscillations in the range of 25–50 Hz convey information important to understand the pathophysiology of diseases relevant to metabolic failure and other abnormal states^8^. Metabolic processes, specifically mitochondrial metabolism, modulate gamma oscillations^32^. Oscillating high frequency power line artifacts can disrupt these frequencies, but they can be reduced by placing the pig inside a Faraday cage.

With some exceptions, anesthetics generally exert neurophysiological and metabolic suppression or modulation. For example, increasing doses (or inhaled fraction) of isoflurane gradually suppress neural activity, with elevated doses leading to EEG burst suppression^29^. Drugs frequently utilized in man such as midazolam have not been as well characterized in animal studies. This is compounded by the practice of combining these drugs with other agents that help induce general anesthesia. Pentobarbital is a common veterinary euthanasia drug. Due to prolonged half-life, it is not a viable anesthesia option for procedures where reversibility is necessary. We found both midazolam and pentobarbital not suitable for our method given the nonlinearity of effects relative to dosages, which was compounded by animal variability. Furthermore, midazolam gives rise to an active or partially active metabolite (OH-midazolam), further introducing variability. In contrast, isoflurane anesthesia is reversible, replicable, and compatible with the recording of activity similar to human electrocorticography. Our results show that only the characteristic alpha frequency peak, a distinct feature of the awake human EEG, is not present in the pig isoflurane anesthetized recordings. In this study, inferences for the anesthetic effects of midazolam and pentobarbital are only qualitative. Quantification will require further studies. Similar considerations apply to the dose dependence of the signals recorded under varying isoflurane concentrations.

Standard multielectrode array insertion into the cortex is a traumatic event that can lead to injury of neurons, blood–brain barrier disruption, localized ischemic stroke or microhemorrhages, and immediate recruitment microglia^33–35^. Unlike the standard microelectrode arrays, the a-SiC ultrasmall microelectrode arrays reported here have a high flexural tolerance that resists fracture^36^ and have a small cross-section, reducing insertion trauma and thus the confounding effects of implantation disruption on local neural activity^37–40^. The a-SiC arrays also better match the mechanical properties of cortical tissue and thus reduce forces on the neural tissue associated with breathing-related brain movements, thus improving recordings^41,42^. Here we showed that the amplitudes of signals recorded, at least within the limits of these studies, can be up to 16 times higher with a-SiC probes than with standard probes, demonstrating that these novel electrodes have advantages for intracortical recordings in the pig.

Future avenues of investigation hereby enabled include the correlation of native pig single neuron activity with large surface area recordings such as ECoG. This should prove helpful in the investigation of network properties not fully dependent on single neuron properties. These properties include the spread of low frequency oscillations via volume conduction, feedforward inhibition at sites remote from lesions, and long range inhibitory neuron activity^43^. Additional uses of this work include the concerted measurement of anesthetic effects on these activities or the simultaneous recording of neuronal ensembles located across the brain under unperturbed and disease states or experimental manipulations.

## Limitations

The use of inbred commercial pigs, which are prone to specific genetic risks^44^ presents a limitation, as perhaps reflected in the two pigs in this study that did not provide data due to neurological diseases. Second, we measured similar ECoG activity in the pig brain compared with the human brain, but did not extend the comparison to human intracortical single-neuron recordings. Lastly, we cannot be sure that craniotomy did not disrupt human or pig ECoG activity.

## Conclusions

The methods described here allow for the preservation of native pig brain neurophysiological activity for several hours. Pig electrocorticography recordings were essentially indistinguishable from awake human recordings except for the small segment of alpha electrical activity associated with vision in conscious persons. In addition, single-neuron and paired-pulse stimulation recordings were feasible simultaneously with electrocorticography and depth electrode recordings. The spontaneous and stimulus-elicited neuronal activities thus surveyed can be recorded with a degree of precision similar to that achievable in rodent or any other animal studies and prove as informative as unperturbed human electrocorticography.

### Supplementary Information

Supplementary Information.

## Data Availability

All data presented or analyzed and the Faraday cage design are available from the corresponding author.
