# Author Correction: Recording of pig neuronal activity in the comparative context of the awake human brain

**DOI:** 10.1038/s41598-022-25156-8

**Published:** 2022-11-29

**Authors:** Aksharkumar Dobariya, Tarek Y. El Ahmadieh, Levi B. Good, Ana G. Hernandez‑Reynoso, Vikram Jakkamsetti, Ronnie Brown, Misha Dunbar, Kan Ding, Jesus Luna, Raja Reddy Kallem, William C. Putnam, John M. Shelton, Bret M. Evers, Amirhossein Azami, Negar Geramifard, Stuart F. Cogan, Bruce Mickey, Juan M. Pascual

**Affiliations:** 1 Rare Brain Disorders Program, Department of Neurology, The University of Texas Southwestern Medical Center, 5323 Harry Hines Blvd. Mail Code 8813, Dallas, TX 75390‑8813 USA; 2 Department of Neurological Surgery, The University of Texas Southwestern Medical Center, Dallas, TX 75390 USA; 3 Department of Bioengineering, The University of Texas at Dallas, Richardson, TX 75080 USA; 4 Animal Resource Center, The University of Texas Southwestern Medical Center, Dallas, TX 75390 USA; 5 Department of Neurology, The University of Texas Southwestern Medical Center, Dallas, TX 75390 USA; 6 Department of Pharmacy Practice and Clinical Pharmacology, Experimental Therapeutics Center, Texas Tech University Health Sciences Center, Dallas, TX 75235 USA; 7 Department of Pharmaceutical Science, School of Pharmacy, Texas Tech University Health Sciences Center, Dallas, TX 75235 USA; 8 Department of Internal Medicine, The University of Texas Southwestern Medical Center, Dallas, TX 75390 USA; 9 Department of Pathology, The University of Texas Southwestern Medical Center, Dallas, TX 75390 USA; 10 Department of Physiology, The University of Texas Southwestern Medical Center, Dallas, TX 75390 USA; 11 Department of Pediatrics, The University of Texas Southwestern Medical Center, Dallas, TX 75390 USA; 12 Eugene McDermott Center for Human Growth and Development/Center for Human Genetics, The University of Texas Southwestern Medical Center, Dallas, TX 75390 USA; 13 Present Address: Department of Neurosurgery, Loma Linda University Medical Center, Loma Linda, CA 92354 USA

Correction to: *Scientific Reports* 10.1038/s41598-022-19688-2, published online 15 September 2022

In the original version of this Article Amirhossein Azami, Negar Geramifard and Stuart F. Cogan were incorrectly affiliated with ‘Department of Pathology, The University of Texas Southwestern Medical Center, Dallas, TX, 75390, USA’. The correct affiliation is listed below.

Department of Bioengineering, The University of Texas at Dallas, Richardson, TX, 75080, USA.

The original Article and accompanying Supplementary Information file have been corrected.

