# Author Correction: Recording of pig neuronal activity in the comparative context of the awake human brain

**DOI:** 10.1038/s41598-024-60111-9

**Published:** 2024-04-29

**Authors:** Aksharkumar Dobariya, Tarek Y. El Ahmadieh, Levi B. Good, Ana G. Hernandez-Reynoso, Vikram Jakkamsetti, Ronnie Brown, Misha Dunbar, Kan Ding, Jesus Luna, Raja Reddy Kallem, William C. Putnam, John M. Shelton, Bret M. Evers, Amirhossein Azami, Negar Geramifard, Stuart F. Cogan, Bruce Mickey, Juan M. Pascual

**Affiliations:** 1 Rare Brain Disorders Program, Department of Neurology, The University of Texas Southwestern Medical Center, 5323 Harry Hines Blvd. Mail Code 8813, Dallas, TX 75390-8813 USA; 2 Department of Neurological Surgery, The University of Texas Southwestern Medical Center, Dallas, TX 75390 USA; 3 Department of Bioengineering, The University of Texas at Dallas, Richardson, TX 75080 USA; 4 Animal Resource Center, The University of Texas Southwestern Medical Center, Dallas, TX 75390 USA; 5 Department of Neurology, The University of Texas Southwestern Medical Center, Dallas, TX 75390 USA; 6 Department of Pharmacy Practice and Clinical Pharmacology, Experimental Therapeutics Center, Texas Tech University Health Sciences Center, Dallas, TX 75235 USA; 7 Department of Pharmaceutical Science, School of Pharmacy, Texas Tech University Health Sciences Center, Dallas, TX 75235 USA; 8 Department of Internal Medicine, The University of Texas Southwestern Medical Center, Dallas, TX 75390 USA; 9 Department of Pathology, The University of Texas Southwestern Medical Center, Dallas, TX 75390 USA; 10 Department of Physiology, The University of Texas Southwestern Medical Center, Dallas, TX 75390 USA; 11 Department of Pediatrics, The University of Texas Southwestern Medical Center, Dallas, TX 75390 USA; 12 Eugene McDermott Center for Human Growth and Development/Center for Human Genetics, The University of Texas Southwestern Medical Center, Dallas, TX 75390 USA; 13 Present Address: Department of Neurosurgery, Loma Linda University Medical Center, Loma Linda, CA 92354 USA

Correction to: *Scientific Reports* 10.1038/s41598-022-19688-2, published online 15 September 2022

The original version of this Article contained errors in the legend of Figure 6.

“Neurophysiological effects of isoflurane. Dose dependent and reversible anesthetic effect of isoflurane general anesthesia on the ECoG. (**A**) Mean and standard error of the power spectral density recorded under 2% isoflurane (black spectra with error lines), 3% isoflurane (red spectra with error lines) and re-adjusted 2% isoflurane (blue spectra with error lines). (**B**) Coherence under 2% isoflurane (black spectra with error lines), 3% isoflurane (red spectra with error lines) and re-adjusted 2% isoflurane (blue spectra with error lines).”

now reads:

“Neurophysiological effects of isoflurane. Dose dependent anesthetic effect of isoflurane general anesthesia on the ECoG. **A**. Mean and standard error of the power spectral density recorded under 1% isoflurane (black spectra with error lines), 2% isoflurane (blue spectra with error lines) and 3% isoflurane (red spectra with error lines). **B**. Coherence under 1% isoflurane (black spectra with error lines), 2% isoflurane (blue spectra with error lines) and 3% isoflurane (red spectra with error lines).”

Additionally, a subheading of the Results section contained an error.

“Dose dependent, reversible effect of isoflurane anesthesia in the pig.”

now reads:

“Dose dependent effect of isoflurane anesthesia in the pig.”

The original Article has been corrected.

