# Supplementary material for: Recording of pig neuronal activity in the comparative context of the awake human brain
Source: Sci Rep. 2022 Sep 15;12:15503. doi: 10.1038/s41598-022-19688-2 (PMC9478131; doi:10.1038/s41598-022-19688-2)
Supplement: Supplementary file 1 — Supplementary Information. [file 41598_2022_19688_MOESM1_ESM.docx]

# SUPPLEMENTARY INFORMATION

**Recording of pig neuronal activity in the**

**comparative context of the awake human brain**

Aksharkumar Dobariya^1, ¶^, Tarek El Ahmadieh ^2, ¶, *^, Levi B. Good ^1^,

Ana G. Hernandez-Reynoso ^3^, Vikram Jakkamsetti ^1^, Ronnie Brown ^2^, Misha Dunbar ^4^,

Kan Ding ^5^, Jesus Luna ^5^, Raja Reddy Kallem ^6^, William C. Putnam ^6, 7^, John M. Shelton ^8^,

Bret M. Evers ^9^, Amirhossein Azami ^3^, Negar Geramifard ^3^, Stuart F. Cogan ^3^, Bruce Mickey ^2^, Juan M. Pascual ^1, 10, 11, 12^

1 Rare Brain Disorders Program, Department of Neurology, 2 Department of Neurological Surgery, The University of Texas Southwestern Medical Center, Dallas, Texas 75390; 3 Department of Bioengineering, The University of Texas at Dallas, Richardson, Texas, 75080; 4 Animal Resource Center, 5 Department of Neurology, The University of Texas Southwestern Medical Center, Dallas, Texas 75390; 6 Department of Pharmacy Practice and Clinical Pharmacology and Experimental Therapeutics Center, 7 Department of Pharmaceutical Science, School of Pharmacy, Texas Tech University Health Sciences Center, Dallas, Texas 75235; 8 Department of Internal Medicine, 9 Department of Pathology, 10 Department of Physiology, 11 Department of Pediatrics, 12 Eugene McDermott Center for Human Growth & Development/ Center for Human Genetics. The University of Texas Southwestern Medical Center, Dallas, Texas 75390, USA.

¶ Equal contribution

* Present address: Department of Neurosurgery, Loma Linda University Medical Center, Loma Linda, CA 92354.

# SUPPLEMENTARY FIGURES


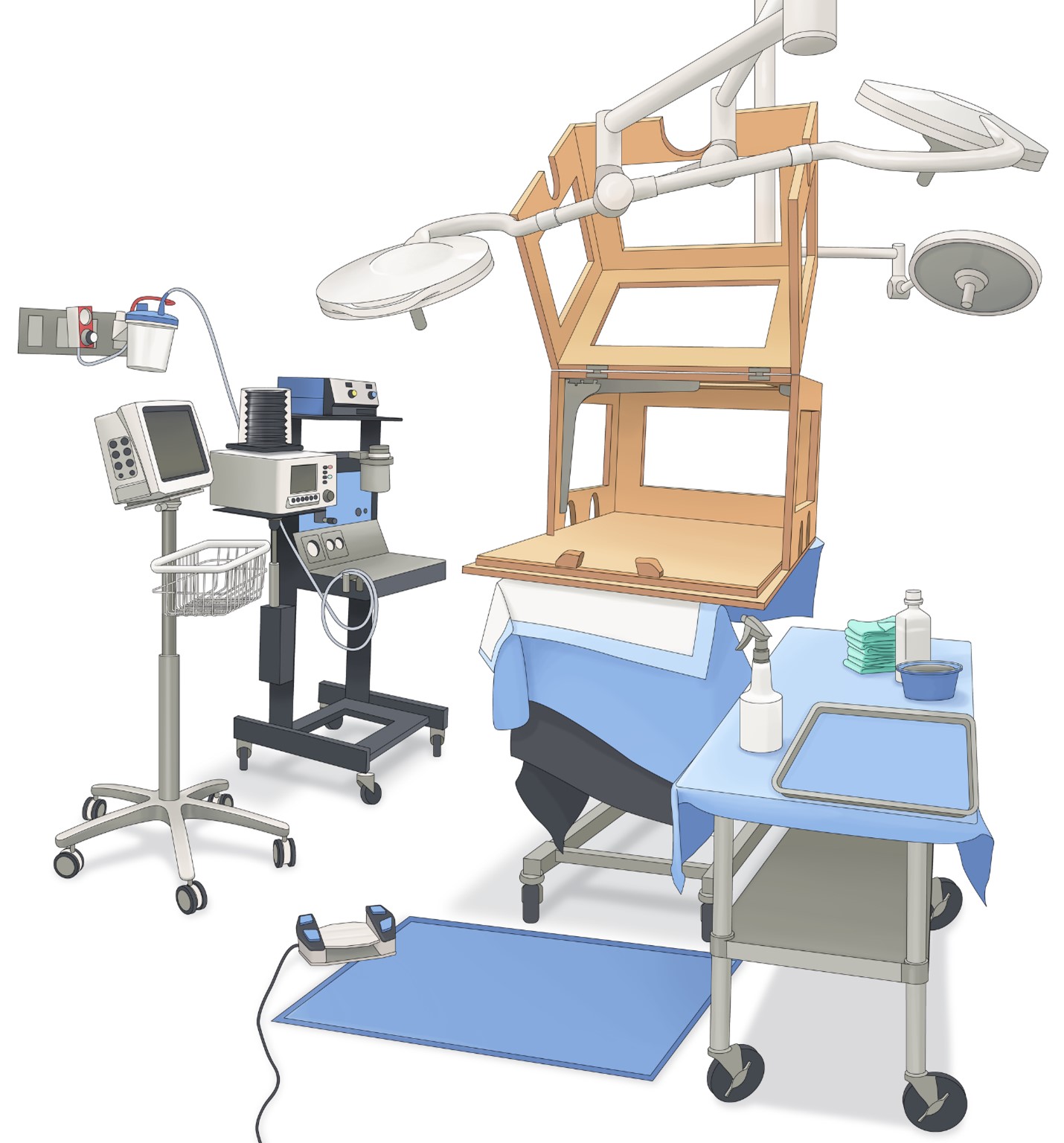


**Supplementary figure 1**. **Simplified operating room configuration**. The figure illustrates the relative position of some of the equipment used for craniectomy and neurophysiological recording. This includes physiological parameter monitors, suction-wall mount anesthesia machine, operating table, Faraday cage, surgical lights, and instrument tray.

**
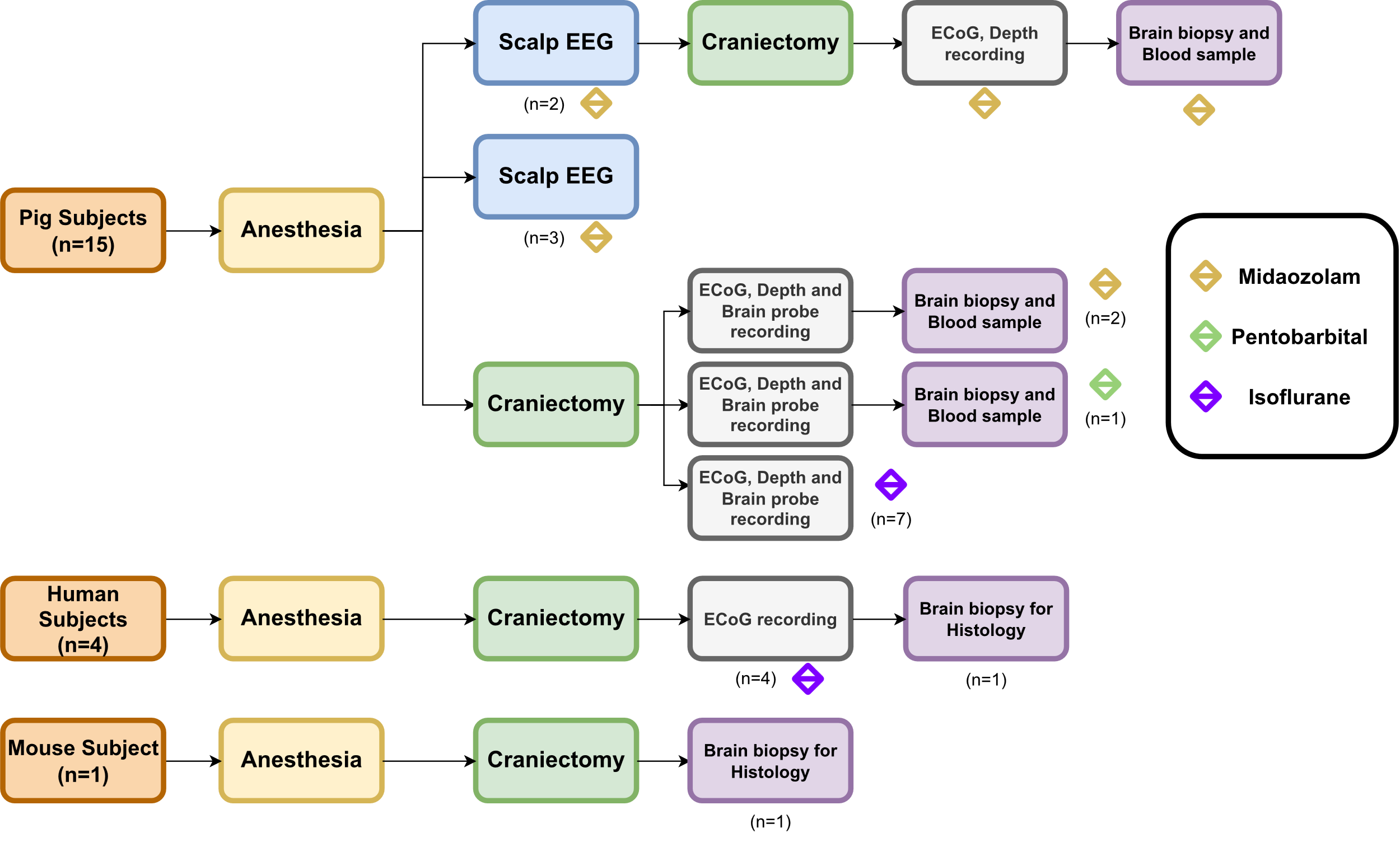
**

**Supplementary figure 2**. **Overview of data sources**. Methods or procedures conducted on all human and animal experimental subjects, including choice of anesthesia.

**
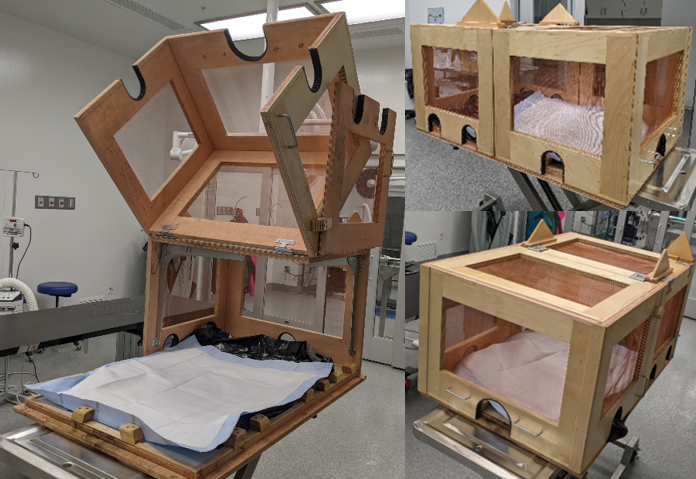
**

**Supplementary figure 3**. **Faraday cage.** This structure is shown in open (left panel) and closed (right panels) configurations.


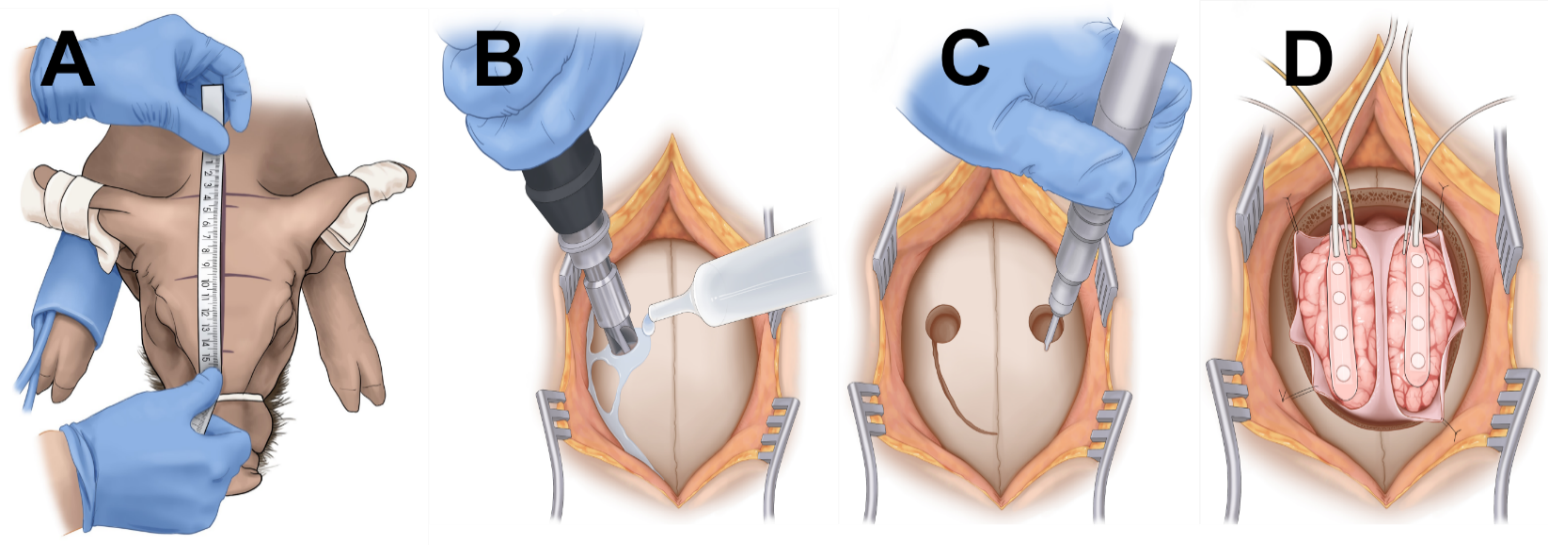


**Supplementary figure 4**. **Craniectomy method and electrode positioning**. **A**. Longitudinal line from 2 cm anterior to eyes and extended posteriorly to the crest of the occipital bone. **B.** Use of a burr hole drill bit and continuous irrigation of saline to make expandable holes. **C**. Craniotome application for the extension of the burr holes. **D**. Exposure of the brain with bilateral 1 × 4 ECoG electrode grid, depth electrodes and brain probe (orange) placement.

**Supplementary figure 5. ECoG analysis sequence.** Power spectral density (PSD) and coherence analysis sequence. NFFT: nonequispaced fast Fourier transform.


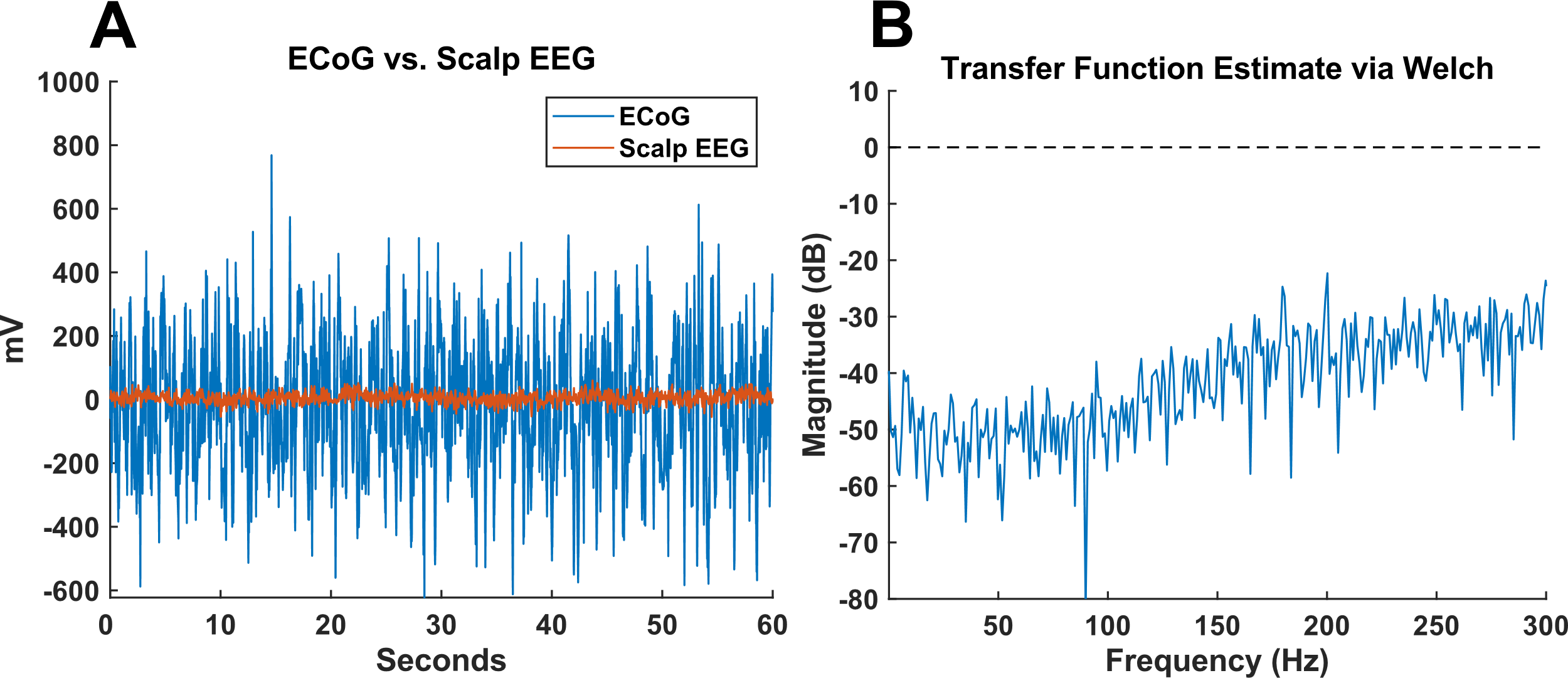


**Supplementary figure 6**. **Comparison of ECoG and scalp EEG signals**. The comparison used a transfer function. **A**. ECoG signal amplitude is several fold larger than that from scalp EEG recordings. **B**. The ECoG filtering or dampening effect is more accentuated for signal frequencies below 100 Hz, gradually decreasing for 100 to 200 Hz, resulting in a plateaued or unform dampening for frequencies greater than 200 Hz measured from scalp EEG recordings.

**
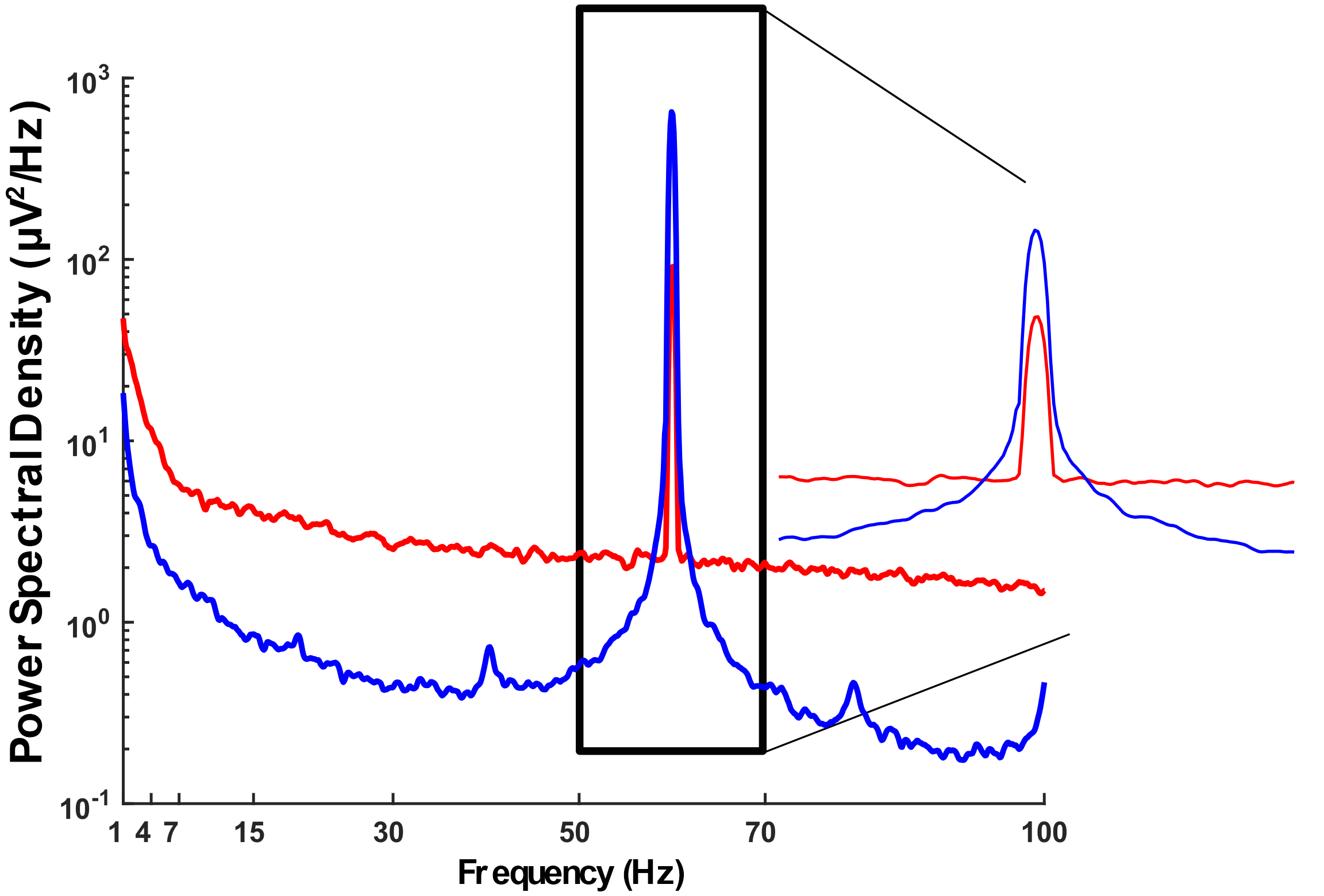
**

**Supplementary figure 7**. **Suppression of electrical noise by the Faraday cage**. Effect on gamma frequencies of the open and closed Faraday cage. The blue line represents power line artifact oscillations that spans the entire gamma range (30-100 Hz). With the Faraday cage closed, the frequency span of the ~60 Hz noise becomes narrower and decreases in amplitude.


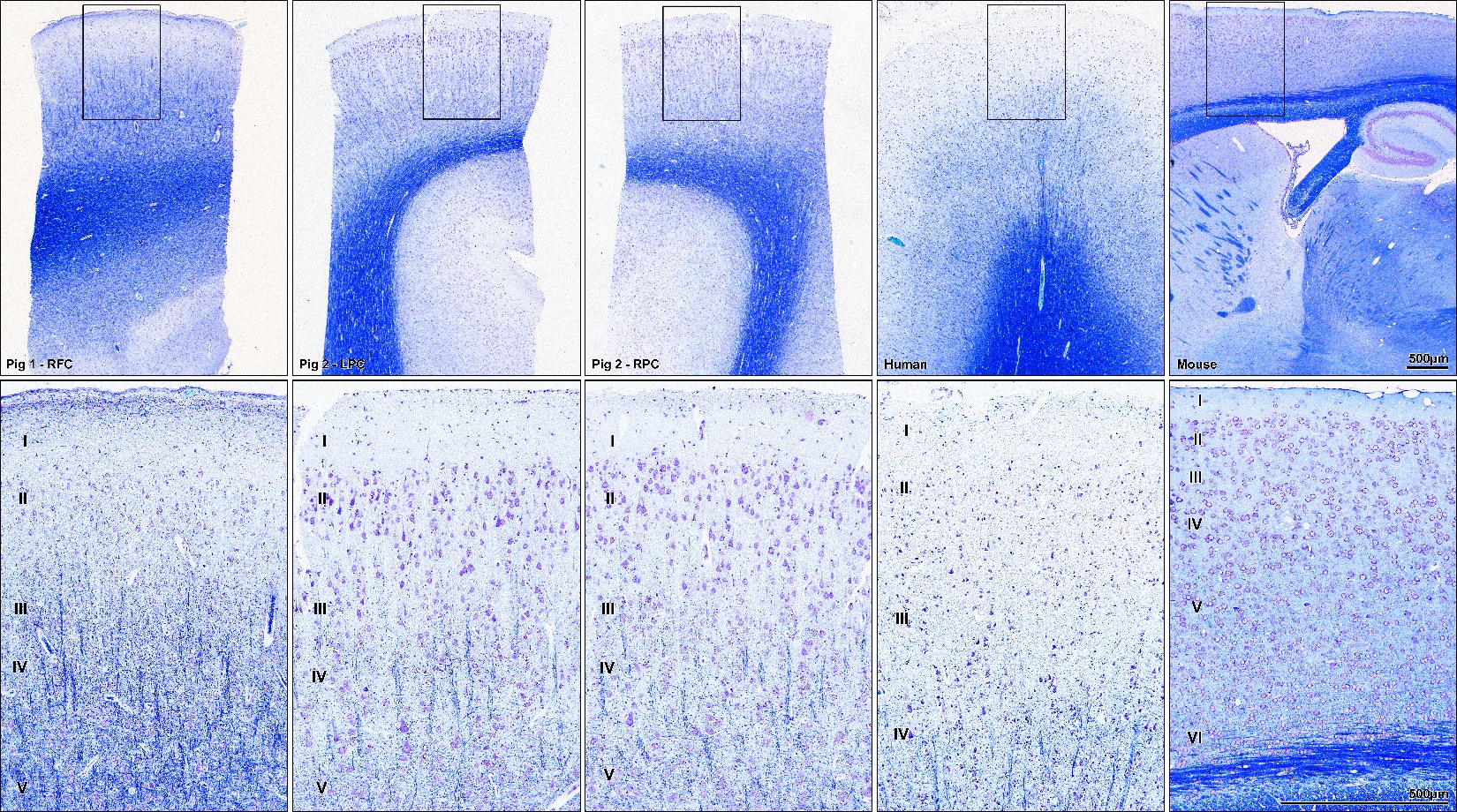


**Supplementary figure 8. Comparative structures of the cerebral cortex.** Representative cortical structure of the porcine, human, and murine frontal lobe stained with Nissl / luxol fast blue. Section thickness: 5 µm, magnification 2.5 × and 10 × objective magnifications; bars of measure equal 500 µm

**
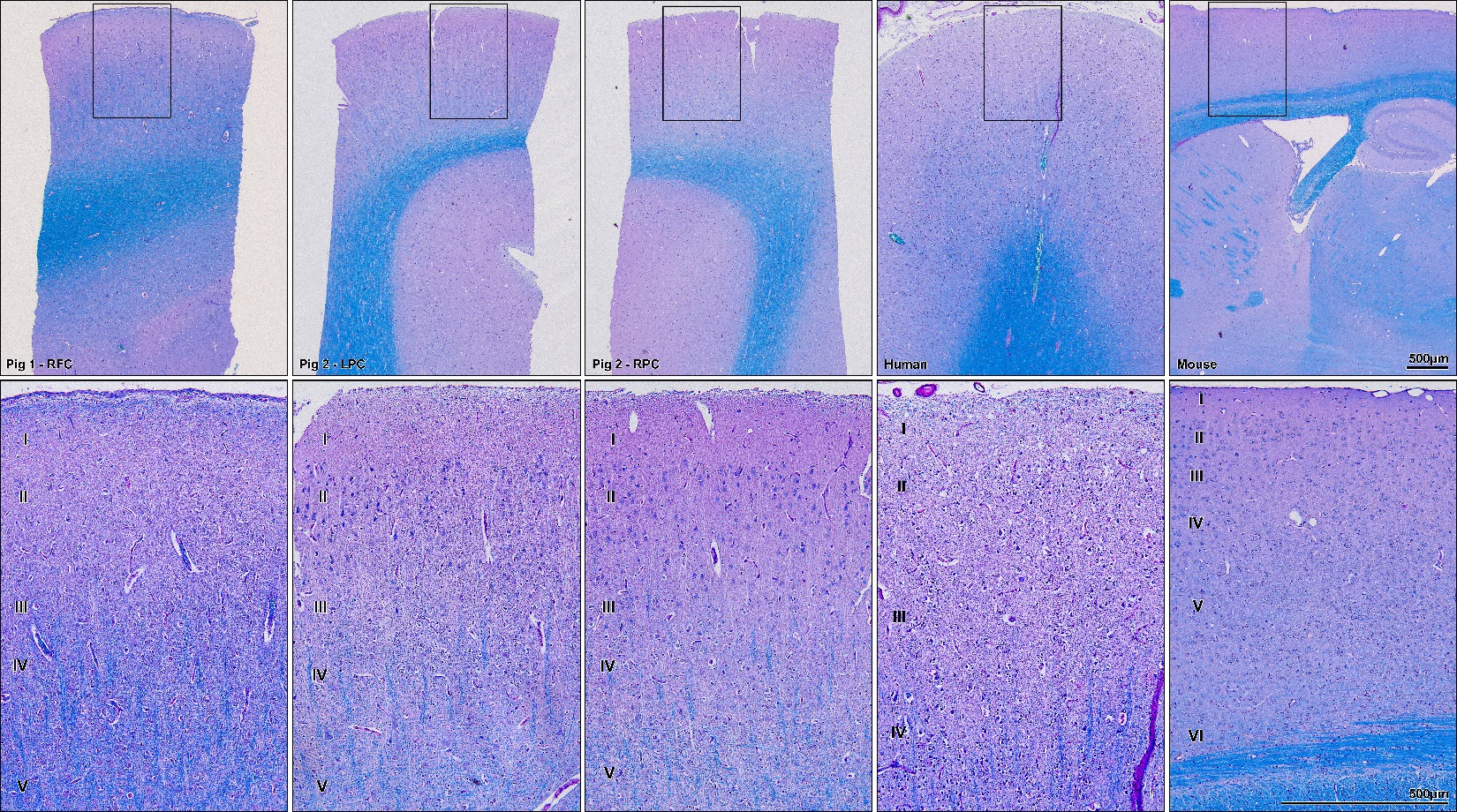
**

**Supplementary figure 9. Comparative structures of the cerebral cortex** (additional staining). Representative cortical structure of the porcine, human, and murine frontal lobe stained with Nissl / Periodic Acid Schiff. Adjacent sections from the previous figure. Section thickness: 5 µm, magnification 2.5 × and 10 × objective magnifications; bars of measure equal 500 µm.

# SUPPLEMENTARY TABLES

| Name | Quantity | Type | Size/Number |
| --- | --- | --- | --- |
| Monopolar cautery | 1 | - | - |
| Towel clamps | 10 | - | - |
| Bayonet forceps | 2 | - | - |
| Penfield dissector | 2 | - | #1 and #3 |
| Rongeurs | 2 | Lecksell Rongeur,  Mastoid Rongeur | - |
| Drill bits | 2 | Perforator drill bit,  Footed attachment  with taper drill bit | - |
| Elevators | 2 | Periosteal elevators | - |
| Needles | 2 | - | #18G and #23G |
| Self-retaining retractors | 2 | Large curved  Weitlaner retractors |  |
| Scissors | 2 | Suture scissors,  Metzenbaum scissors |  |
| Suction tubes | 1 | Frazier suction |  |

**Supplementary table 1.** Surgical instruments used for incision, craniectomy and duramater opening.

| Parameter | Value |
| --- | --- |
| Low-cut filter | 1 Hz |
| High-cut filter | 50 Hz |
| Notch filter | ON |
| Time constant | 0.3 s |
| Sensitivity | 50 µV/division |

**Supplementary table 2.** ECoG recording parameters.

| Subject type | Type of electrode | Data type | Contact area (mm^2^) | Impedance |
| --- | --- | --- | --- | --- |
| Pig | Needle electrode | Eeg | 4.8 | < 10 kohms |
| Pig | Ecog electrode | Ecog | 4.15 | < 2 kohms |
| Pig | Depth electrode | Depth | 8.33 | < 3 - 5 kohms |
| Human | Human ecog electrode | Ecog | 4.15 | < 1.5 kohms |

**Supplementary table 3**. Types of electrodes used and their impedances.

| **Pig** | **Studies** | **Anesthesia** | **Anesthetic level** |
| --- | --- | --- | --- |
| **1** | Scalp EEG | Midazolam | 0.5 mg/Kg/h |
| **2** | Scalp EEG | Midazolam | 0.5 mg/Kg/h |
|  | ECoG | Midazolam | 1 mg/Kg/h |
|  | ECoG | Midazolam | 2 mg/Kg/h |
| **3** | Scalp EEG | Midazolam | 0.5 mg/Kg/h |
| **4** | Scalp EEG | Midazolam | 0.5 mg/Kg/h |
| **5** | Scalp EEG | Midazolam | 0.5 mg/Kg/h |
|  | ECoG | Midazolam | 0.5 mg/Kg/h |
|  | ECoG | Midazolam | 1 mg/Kg/h |
|  | ECoG | Midazolam | 2 mg/Kg/h |
|  | ECoG | Midazolam | 3 mg/Kg/h |
| **6** | Only Brain tissue and plasma midazolam concentration measurement. | | |
| **7** | ECoG & Depth | Midazolam | 2 mg/Kg/h |
|  | ECoG & Depth | Midazolam | 3 mg/Kg/h |
| **8** | ECoG & Depth | Pentobarbital | 20 mg/Kg/h |
|  | ECoG & Depth | Pentobarbital | 40 mg/Kg/h |
|  | ECoG & Depth | Pentobarbital | 60 mg/Kg/h |
| **9** | ECoG & Depth | Isoflurane | 1% |
|  | ECoG & Depth | Isoflurane | 1.50% |
|  | ECoG & Depth | Isoflurane | 3% |
| **10** | Data recorded due to presence of tumor. | | |
| **11** | ECoG & Depth | Isoflurane | 1.50% |
|  | ECoG & Depth | Isoflurane | 2% |
|  | ECoG & Depth | Isoflurane | 3% |
| **12** | Data not recorded due to presence of epileptic events. | | |
| **13** | ECoG & Depth | Isoflurane | 1% |
|  | ECoG & Depth | Isoflurane | 2% |
|  | ECoG & Depth | Isoflurane | 3% |
| **14** | ECoG & Depth | Isoflurane | 1.50% |
| **15** | ECoG & Depth | Isoflurane | 1.50% |
| **16** | ECoG, Depth & Intracortical | Isoflurane | 1.50% |
| **17** | ECoG, Depth & Intracortical | Isoflurane | 1% |

**Supplementary table 4**. **Data obtained from each pig and anesthesia used**. The brain probe measures tissue temperature, barometric pressure and oxygen partial pressure.

## SUPPLEMENTARY CODE 1

## Power spectral density code for MATLAB

clc

close all

clear variables

cd C:\ %data_file_path;

load('****.mat'); %specify the filename

ranges=[0.1 4;3.99 7;6.99 15;14.99 30;29.99 50;0.1 50]; %defining ranges for the EEG frequency components

trace_length=5; % Variable for 5 seconds

sampfreq=1000; % data sampling rate

% Device matrix into 5 second segments & arranging them in columnar sashion

% in new matrix

total_5s_segments=length(data)/(5*sampfreq);

new_matrix=reshape(data,(trace_length*sampfreq),total_5s_segments);

% Subtracting a mean of columns from a data matrix

mean_matrix=new_matrix-mean(new_matrix,1);

% Calculating NFFT points

num_bins=size(ranges,1)-1;

num_total=size(ranges,1);

data_length=floor(sampfreq*trace_length)+1;

NFFT = 2^nextpow2(data_length);

% FFT of 5 second segment data from NFFT points

fft = fft(mean_matrix,NFFT,1)/data_length;

% Frequency row vector

freq = sampfreq/2*linspace(0,1,NFFT/2+1);

% Dividing fft into half

vals1=2*abs(fft(1:NFFT/2+1,:));

% Calculating the relative power

yvals1=vals1(freq>ranges(size(ranges,1),1) & freq<ranges(size(ranges,1),2),:);

xvals1=freq(freq>ranges(size(ranges,1),1) & freq<ranges(size(ranges,1),2));

total_power=trapz(xvals1,yvals1);

vals3=vals1./total_power;

% Averaging the absolute and raw powers

avg_spect=mean(vals1,2);

avg_spect_rel=mean(vals3,2);

% Plotting data for absolute power, follow similar code for relative and just change the variables.

figure(1),clf,hold on,

plot(freq,avg_spect,'b')

set(gca,'xscale','log','yscale','log','xlim',[1 50],'xtick',[1 4 7 15 30 40 50],'xticklabel',[1 4 7 15 30 40 50])

xlabel('Frequency (Hz)')

ylabel('Power Spectral Density (uV^2/Hz)')

title('Power Spectral Density Spectra');

legend('R1');

## SUPPLEMENTARY CODE 2

## Coherence Code for MATLAB

clc

close all

clear variables

cd C:\ %data_file_path;

load('****.mat'); %specify the filename

ranges=[0.1 4;3.99 7;6.99 15;14.99 30;29.99 50;0.1 50]; %defining ranges for the EEG frequency components

trace_length=5; % Variable for 5 seconds

sampfreq=1000; % data sampling rate

% Device matrix into 5 second segments & arranging them in columnar sashion

% in new matrix for both sides

total_5s_segments=length(L1)/(5*sampfreq);

matrix_left=reshape(L1,(trace_length*sampfreq),total_5s_segments);

matrix_right=reshape(R1,(trace_length*sampfreq),total_5s_segments);

% Subtracting a mean of columns from a data matrix for both sides

mean_left_matrix=matrix_left-mean(matrix_left,1);

mean_right_matrix=matrix_right-mean(matrix_right,1);

% Calculating NFFT points

data_length=floor(sampfreq*trace_length)+1;% floor rounds the value of variable to an integer place on the minus infinity side, i.e. -2.5 will be -3 and 5.6 will become 5.

NFFT = 2^nextpow2(data_length);

nfft=2000;

layer=[];

% Using mscohere, calculating coherence

[Cxy, F] = mscohere(mean_left_matrix,mean_right_matrix,hamming(round(sampfreq)/5),round(sampfreq/10),nfft,round(sampfreq));

coherence1=[];

% Row-wise mean of coherence data

coheretemp1=nanmean(Cxy,2);

% Plotting data

figure(1),clf,hold on

plot(F,coheretemp1,'b')

set(gca,'xlim',[0 50],'ylim',[0 1])

xlabel('Frequency (Hz)')

ylabel('Coherence')

title('Coherence spectrum across frontal nodes (L1 – R1)')
